# Mitochondrial DNA control region sequencing of the critically endangered Hainan gibbon (*Nomascus hainanus*) reveals two female origins and extremely low genetic diversity

**DOI:** 10.1080/23802359.2021.1909432

**Published:** 2021-04-07

**Authors:** Yanqing Guo, Dong Peng, Ling Han, Tao Liu, Gang Li, Paul A. Garber, Jiang Zhou

**Affiliations:** a College of Life Sciences, Northwest University, Xian, China; b School of Karst Science, Guizhou Normal University, Guiyang, China; c Department of Anthropology, Program in Ecology, Evolution, and Conservation Biology, University of Illinois, Urbana, IL, USA; d International Centre of Biodiversity and Primate Conservation, Dali University, Dali, Yunnan, China

**Keywords:** Genetic diversity, mitochondrial DNA, Hainan Gibbon

## Abstract

The Hainan gibbon (*Nomascus hainanus*) is endemic to China and is the world’s rarest ape. The remaining wild population totals only 33 individuals. In the current study, we sequenced the Mitochondrial DNA control region of 12 wild Hainan gibbons representing three social groups of the five remaining groups. By conducting population genetic analyses, we found that the proportion of four nucleotides (T, C, A and G) were 29.0%, 27.2%, 31.9% and 11.9%, respectively. Hypervariable segments of the mtDNA D-loop region (1005 bp in length), indicated five variable sites (a point mutation), with only two haplotypes present among the 12 samples. We observed that the genetic diversity of Hainan gibbons is lower than that reported in any other wild primate population, and that the two haplotypes detected, represent two ancestral lineages. These findings have important implications for proposing effective conservation strategies to protect this Critically Endangered ape species.

## Introduction

The Hainan gibbon (*Nomascus hainanus,* Hylobatidae, Primates) (Thomas 1892) is the world’s rarest ape, with a remaining wild population of some 33 individuals. Although once widespread across Hainan, China, the last remining population is confined to a forested area of 16 km^2^ in the Bawnagling National Nature Reserve. Recent estimates indicate the existence of five remaining social groups (A, B, C, D, E) (Unpublished data from 2020 monitoring results). Hainan gibbons experienced a rapid population decline in the mid 20th century, as 99.9% of their habitat was lost due to the conversion of tropical forests for purposes of industrial agriculture. In the 1970s, the Hainan gibbon population totaled only 7–8 individuals (Liu et al. 1984; Zhou et al. 2005; Deng et al. 2015). And, although the population has increased fourfold over the past 40 years, this species continues to face an impending extinction crisis.

Population genetics offer an important tool to better understand and model the evolutionary history, population dynamics, and patterns of gene flow of threatened species. This information is essential in developing effective strategies to manage and protect wild animal populations. For example, studies have shown that over the past few centuries, orangutans (*Pongo pygmaeus)* inhabiting northeastern Borneo have experienced a genetic bottleneck associated with a population decline of some 95%. This has occurred in response to anthropogenically induced habitat fragmentation, deforestation, and hunting (Goossens et al. 2006). Similarly, the Mexican howler monkey (*Alouatta palliata mexicana),* an Endangered primate subspecies is currently distributed in only four forest fragments in the state of Veracruz, Mexico. Genetic testing revealed that haplotype diversity and nucleotide diversity (h = 0.486; π = 0.0007) are extremely low compared with other Neotropical primates (Jacob et al. 2014). Finally, in northwestern Madagascar, populations of the golden-brown mouse lemur (*Microcebus ravelobensis*) have been severely reduced, resulting in a dramatic decrease in genetic diversity (Guschanski et al. 2007). Although the set of anthropogenic factors that promote wildlife population decline and biodiversity loss are well understood (Estrada et al. 2018; Estrada et al. 2019; Estrada et al. 2020), the specific effects of population decline on genetic diversity and extinction risk in individual taxa require continued investigation.

In the current study we examine levels of genetic diversity in the mitochondrial DNA (mtDNA) control region of the Critically Endangered Hainan gibbon. Previous research on the genetics of this species have focused principally on their phylogenetic position within the gibbon radiation (Su et al. 1995; Zhang 1995; Thinh et al. 2010a,b). To date, there has been only one study of the genetic diversity of the mitochondrial D-loop region of the Hainan gibbon (Li et al. 2010). However, given that the genetic data used in that study came from a single family group and focused on a short segment (202 bp) of the D-loop, it is unlikely to fully reflect the range of genetic variability of this Critically Endangered species.

## Methods

### Sampling collection

We studied *Nomascus hainanus* at the Bawangling National Nature Reserve (19 N 02′～19 N 08′, 109 E 02′～109 E 13′), Hainan, China. Hainan gibbons vocalize almost every morning, and we monitored these vocalizations to identify group location (this was done for groups A, B, C, and D; we did not monitor group E). The total population size of the four monitored groups was 25 individuals.

Hainan gibbons are strictly arboreal and often travel in the uppermost regions of the tree canopy. Therefore, collecting noninvasive fresh fecal samples from all group members requires months of intense field observations. From March through August 2018, we spent five months following gibbon groups A, B, and C in order to obtain fresh fecal samples. Gibbon feces noticeably change color approximately two hours after defecation. We used this color change to only collect recently voided fecal samples (samples voided in the previous two hours). To avoid resampling the same individual, each fecal sample was scored for freshness (color), size, and shape. In those instances in which two or more fecal samples were located within a radius of 1.5 m, only one sample was collected. We used high temperature sterilized tweezers and petri dishes to collect the fecal samples. The samples were stored in liquid nitrogen, and then packed in dry ice and transported to the laboratory for cryogenic storage.

### DNA extraction and detection

The average weight of a Hainan gibbon fecal clump is approximately 1000 mg. After carefully examining the fecal clump and judging it to be fresh, we extracted a 100–150mg sample from its interior or center. We extracted DNA from this 100–150mg sample using a QIAamp Fast DNA Stool Mini Kit following the manufacturer’s instructions. We avoided cross-contamination by using an ultra-clean workbench for extracting fecal genomic DNA. The extracted total DNA was subjected to 0.8% agarose gel electrophoresis, and contamination was monitored by including a negative extraction control (mock extraction submitted to PCR) per extraction. GreenView nucleic acid dye staining, and the estimated concentration and purity (260/280, 260/230 value) were recorded using a UV transilluminator. At the same time, we used a Qubit3.0 fluorescence quantifier to determine the concentration of DNA. In order to detect whether the extracted DNA concentration met the standard (≥50 ng/uL), we combined agarose gel electrophoresis, nucleic acid dye staining, and included a fluorescence quantifier to make to increase the reliability of the results.

### Identification of polymorphic markers

#### Testing Potential Markers via amplification

We tested seven hypervariable segments of the Mitochondrial D-loop region primer pairs previously described as polymorphic (Table 1). Samples were amplified for each primer pair via PCR in a reaction volume of ∼10 uL containing 1 uL template DNA (≥50 ng/uL), 0.5 uL (10 pmol/uL) primer, 1 uL bovine serum albumin (New England BioLabs), 2 ul ddH_2_O, and 5 uL PCR Mix. The thermal profile for PCRs consisted of the following: denaturation and enzyme activation at 94 °C (3 min), 35 cycles of denaturation at 94 °C (30 s), annealing at 46–55 °C (30 s), extension at 72 °C (60 s) and final extension at 72 °C (10 min). PCR products were separated on 3% agarose gels by electrophoresis to visually assess the amplification efficiency, and set a negative PCR control in order to ensure amplifications were executed for Mitochondrial D-loop region of Hainan gibbon.

**Table 1.** Mitochondrial D-Loop region primer information.

Primer name	Primer sequence (5′-3′)	bp	Reference
GDL-L1 (F)	CGAAAACAAAATACTCAAATGAACCT	750	Kocher et al. (1989)
GDL-H2 (R)	GGTGATCCATCGTGATGTCTTATT	750	Kocher et al. (1989)
GIBDLF3 (F)	CTTCACCCTCAGCAC CCAAAG C	600	Cummins (2001)
GIBDLR4 (R)	GGGTGATAGGCCTGTGATC	600	Cummins (2001)
L15997 (F)	CACCATTAGCACCCAAAGCT	500	Andayani et al. (2001)
H16498 (R)	CCTGAAGTAGGAACCAGATG	500	Andayani et al. (2001)
L16007 (F)	CCCAAAGCTAAAATTCTAA	450	Whittaker et al. (2007)
H16431 (R)	GTTGGTGATTTCACGGAGGA	450	Whittaker et al. (2007)
L16205 (F)	AACACAACATGCTTACAAGC	1000	Chan et al. (2007)
H16431 (R)	GTTGGTGATTTCACGGAGGA	1000	Chan et al. (2007)
L15926 (F)	TCAAAGCTTACACCAGTCTTGTAAACC	200	Li et al. (2010)
H00651 (R)	TAACTGCAGAAGGCTAGGACCAAACCT	200	Li et al. (2010)
L16007 (F)	CCCAAAGCTAAAATTCTAA	1000	Kuebler et al. (2016)
H00651 (R)	TAACTGCAGAAGGCTAGGACCAAACCT	1000	Kuebler et al. (2016)

F: Forward primer; R: Reverse primer

### Determination and proofreading of the target sequences

One pair of primers with the highest amplification rate and the longest fragment was selected from the initial 7 primer pairs and used for final target sequencing. The PCR products were sent for bidirectional sequencing to Kinco Biotech. Sequencing was performed on the ABI3730XL sequencer using the BigDye® Terminator v3.1 Cycle Sequencing Kit. The sequencing results were aligned in the GenBank sequence database using Blast software, in order to confirm that the amplification primer matched the target sequence. We used Clustal X (1.83) (Jeanmougin et al. 1998) for sequences alignment.

### Data analysis

We evaluated the genetic diversity of the Hainan gibbon population by calculating Haplotype diversity (h) and Nucleotide diversity (π) using *DnaSP* 5.10 (Rozas and Rozas 1999). We assessed base composition and site variation using *MEGA X* (Kumar et al. 2001).

## Results

### DNA sample and identification of polymorphic markers

A total of 36 samples of Hainan gibbon feces were collected, six from Group A, 11 from Group B, and 19 samples from Group C (Supplementary Table S1). Excluding repeated samples (individual recognition results based on microsatellite markers, Guo et al. 2020) and unsuccessfully extracted DNA (<50 ng/uL), the 12 DNA samples used for PCR amplification included three individuals from Group A, four individuals from Group B, and five individuals from Group C.

**Figure 1.**
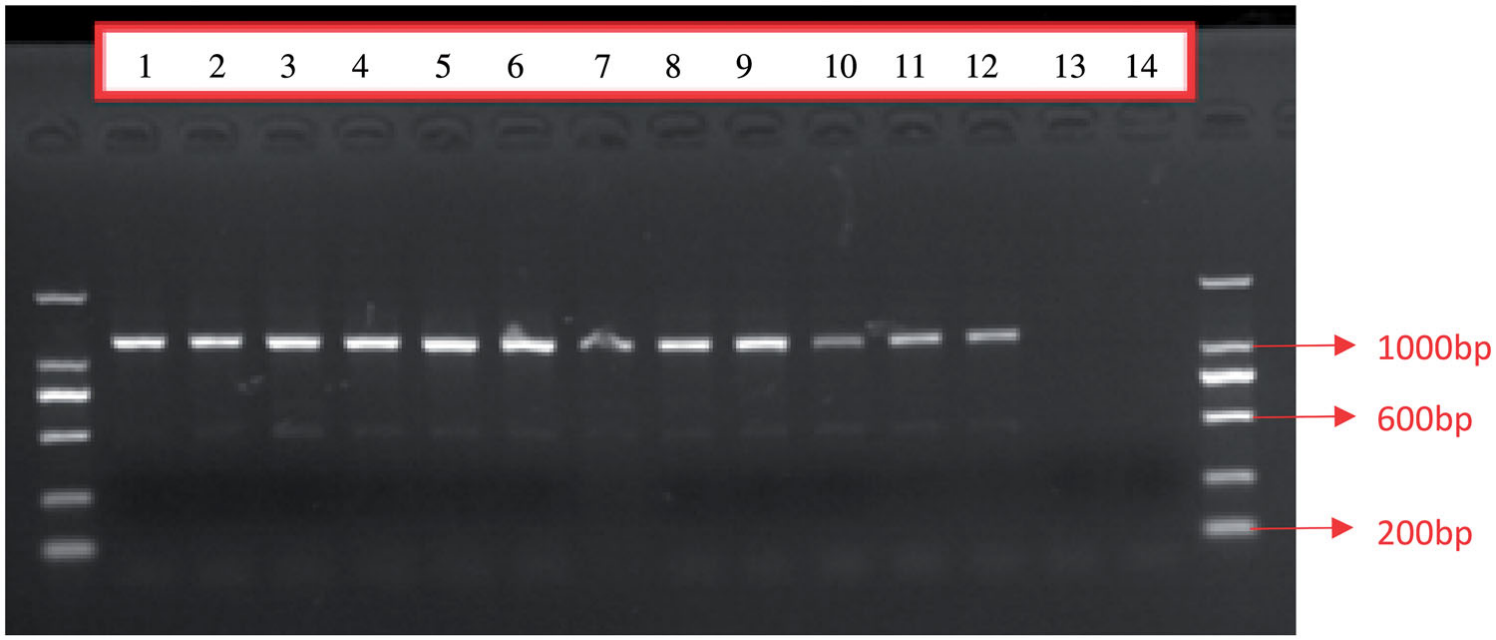
Electrophoretic detection of PCR products in the mtDNA D-Loop region from 12 individual Hainan gibbons (1 to 12 is the experimental group, 13 and 14 are negative controls).

According to the success rate of amplification and the completeness of sequence information in the D-loop region and the sequencing results, primers L16007 and H00651 were selected. The PCR products were subjected to 1.5% agarose gel electrophoresis and a clear band (about1005 bp) was obtained (Figure 1).

### Haplotype distribution

**Figure 2.**
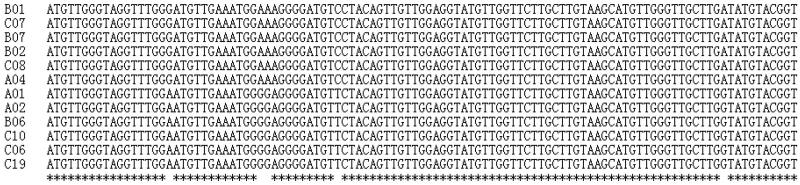
mtDNA D-loop region sequence variation sites in 12 Hainan gibbons.

All 12 sequences defined two distinct haplotypes. Individuals A01, A02, B06, C10, C06, and C19 shared a single haplotype, and individuals B01, C07, B07, B02, C08, and A04 shared a second haplotype. In the 12 samples, five polymorphic sites were detected. Nucleotide variation included conversions, transversions, insertions and deletions. Nucleotide polymorphic sites were primarily G to A conversions. The average content of T, C, A, and G bases in all sequences was 29.0%, 27.2%, 31.9% and 11.9%, respectively (Figure 2).

### Genetic diversity

The haplotype diversity (h) was 0. 545 and the nucleotide diversity (π) was 0. 000271.

## Discussion

In the face of climate change, habitat degradation, forest fragmentation, and the conversion of natural landscapes for purposes of industrial agriculture to feed a growing human population, many animal species have experienced marked population decline (Estrada et al. 2020). A recent report of the United Nations Intergovernmental Science-Policy Platform on Biodiversity and Ecosystem Services estimated that one million animal and plant species are threatened with extinction (IPBES 2019), and this includes some 65% of the over 500 species of nonhuman primates (Estrada et al. 2017; IUCN 2020). In order to better understand the population demography and population genetics of the Critically Endangered Hainan gibbon, our research team first conducted a long-term field investigation monitoring their behavior, ecology, group size, and group composition. In the present study we collected fresh fecal samples of 60% of the members of Group A (n = 3), 57% of the members of Group B (n = 4), and 50% of the membership of Group C (n = 5). The proportion of individuals sampled in our study is larger than in previous studies（Li et al. 2010; Bryant et al. 2016). In addition, we amplified the 1005 bp sequence of the mtDNA D-loop region, which is considerably longer than the 202 bp sequence analyzed in the only other study of mtDNA in this species (Li et al. 2010). Using a longer sequence is expected to improve the reliability of the Hainan gibbon genetic information.

The molecular markers identified in the mtDNA D-loop region of the last remaining population of Hainan gibbons suggest that the two haplotypes detected represent two ancestral female lineages. In response to rapid population decline and exceptionally small population size, the genetic diversity of this ape population remains extremely low. (Gibbs 2001; Püttker et al., 2008; Koskimäki et al. 2014).

This study represents the most comprehensive investigation of the genetic status of this rarest ape population. Our results indicate extremely low levels of polymorphism compared with available data on other wild primate populations, it also is among the lowest genetic variability reported for any highly isolated animal population (Newman et al. 2004; Hayaishi and Kawamoto 2006; Li et al. 2007; Liu et al. 2007; Li et al. 2010; Zhu et al. 2016). The low genetic diversity of the remaining Hainan gibbon population is consistent with their severe population decline (99.4%), that occurred over a 20–30 year period (Zhou 2008), which was the result of extreme deforestation and forest fragmentation that decreased their remaining area of suitable habitat from 27,784 km^2^ (Zhou et al. 2005) to approximately 16 km^2^ (Zhou 2008). The Hainan gibbon remains at extreme risk of extinction. Our findings reinforce the imperative to expand conservation efforts to protect the world’s last remaining Hainan gibbon population. We recommend a targeted conservation program of continuous population monitoring, regenerating native forests, strict enforcement against hunting, and obtaining genetic information on all remaining wild individuals. In the absence of an aggressive and comprehensive conservation management program, this rarest of ape species may not survive to the end of this century.

## Supplementary Material

Supplemental Material

## Data Availability

The data that support the findings of this study are openly available in NCBI at https://www.ncbi.nlm.nih.gov/, reference number [MW052603, MW052604, MW052605, MW052606, MW052607, MW05260, MW052609, MW052610, MW052611, MW052612, MW052613, MW052615].
