# Supplementary material for: Mitochondrial DNA control region sequencing of the critically endangered Hainan gibbon (Nomascus hainanus) reveals two female origins and extremely low genetic diversity
Source: Mitochondrial DNA B Resour. 2021 Apr 7;6(4):1355–9. doi: 10.1080/23802359.2021.1909432 (PMC8032330; doi:10.1080/23802359.2021.1909432)
Supplement: Supplemental Material [file TMDN_A_1909432_SM0810.docx]

S1 Faecal sample collection information

| Group A | | | |
| --- | --- | --- | --- |
| Samples | Date | Local | Individual information |
| 1 | 2017.05.18 | Daankou | Adult female |
| 2 | 2017.06.27 | Honghegu | Juvenile male |
| 3 | 2017.06.27 | Honghegu | unknown |
| 4 | 2017.06.27 | Honghegu | Adult male |
| 5 | 2017.06.27 | Honghegu | unknown |
|  | 2017.06.27 | Honghegu | Adult female |
| Group B | | | |
| Samples | Date | Local | Individual information |
| 1 | 2017.06.03 | Hengganggou | Adult female |
| 2 | 2017.06.03 | Hengganggou | Adult male |
| 3 | 2017.06.04 | Nanbangou | Subadult |
| 4 | 2017.06.04 | Nanbangou | unknown |
| 5 | 2017.06.04 | Nanbangou | Subadult |
| 6 | 2017.06.05 | Nanbangou | Adult male |
| 7 | 2017.06.05 | Nanbangou | Juvenile male |
| 8 | 2017.06.05 | Nanbangou | Adult female |
| 9 | 2017.06.05 | Nanbangou | Juvenile male |
| 10 | 2017.06.05 | Nanbangou | unknown |
| 11 | 2017.06.05 | Nanbangou | unknown |
| Croup C | | | |
| Samples | Data | Local | Individual information |
| 1 | 2017.02.18 | Miaocun | Subadult |
| 2 | 2017.02.18 | Miaocun | Adult male |
| 3 | 2017.02.18 | Miaocun | Adult female |
| 4 | 2017.02.28 | Miaocun | unknown |
| 5 | 2017.03.03 | Miaocun | Subadult |
| 6 | 2017.03.03 | Miaocun | Sub-adult |
| 7 | 2017.03.03 | Miaocun | Sub-adult |
| 8 | 2017.03.03 | Miaocun | Adult female |
| 9 | 2017.03.03 | Miaocun | unknown |
| 10 | 2017.03.03 | Miaocun | Adult female |
| 11 | 2017.03.03 | Miaocun | unknown |
| 12 | 2017.03.03 | Miaocun | unknown |
| 13 | 2017.03.03 | Miaocun | unknown |
| 14 | 2017.03.03 | Miaocun | unknown |
| 15 | 2017.03.06 | Miaocun | unknown |
| 16 | 2017.03.06 | Miaocun | unknown |
| 17 | 2017.03.06 | Miaocun | unknown |
| 18 | 2017.03.07 | Miaocun | unknown |
| 19 | 2017.03.07 | Miaocun | Sub-adult |

Unknown: The individual source of the feces sample is unclear.
